# Patients' Perception of the Use of the EasyPod™ Growth Hormone Injector Device and Impact on Injection Adherence: A Multi-Center Regional Study

**DOI:** 10.3389/fped.2022.839278

**Published:** 2022-02-28

**Authors:** Asma Deeb, Saif Al Yaarubi, Bassam Bin Abbas, Jamal Al Jubeh, Deepti Chaturvedi, Noura Al Hassani, Angham Mutair, Neamat Al Masri, Yazan Al Sanad, Azza Al Shidhani, Noha Samir Mahmoud, Abdullah Alherbish, Martin O. Savage

**Affiliations:** ^1^Sheikh Shahbout Medical City & Khalifa University, Abu Dhabi, United Arab Emirates; ^2^College of Medicine, Sultan Qaboos University Hospital, Muscat, Oman; ^3^King Faisal Specialist Hospital and Research Center, Riyadh, Saudi Arabia; ^4^Department of Pediatrics, Sheikh Khalifa Medical City, Abu Dhabi, United Arab Emirates; ^5^Department of Pediatrics, Burjeel Hospital, Abu Dhabi, United Arab Emirates; ^6^Division of Endocrine and Diabetes, Department of Pediatrics, Tawam Hospital and Faculty of Medicine and Health Science, UAE University, Al Ain, United Arab Emirates; ^7^Pediatric Endocrine Division, Department of Pediatrics, King Abdulaziz Medical City, King Abdullah Specialist Children Hospital, Ministry of National Guard Health Affairs, Riyadh, Saudi Arabia; ^8^Alhabib Medical Group, Riyadh, Saudi Arabia; ^9^Centre for Endocrinology, Barts and the London School of Medicine & Dentistry, William Harvey Research Institute, Queen Mary University of London, London, United Kingdom

**Keywords:** EasyPod™, growth hormone deficiency, recombinant human growth hormone, injector device features, compliance

## Abstract

**Objective:**

This study aimed to assess patient perceptions of the use of the EasyPod™ growth hormone delivery device and its association with compliance.

**Methods:**

This cross-sectional, multicenter study was conducted in six centers from three countries (United Arab Emirates, Oman, and Saudi Arabia,) between March 2020 and June 2020. Children and adolescents aged 3–18 years, diagnosed with growth disorders and receiving rhGH through the EasyPod™ device were enrolled. Patients and caregivers were given a pre-set questionnaire that evaluated patient satisfaction, preference for technical and personalized features, and device drawbacks. The results were analyzed using independent measures of analysis of variance to evaluate the association of higher satisfaction with device features and better compliance.

**Results:**

A total of 186 patients were enrolled in the study. Of these, 45.7% had GH deficiency. The mean age (±SD) of patients was 11.8 (±2.76) years; 117 (62.90%) were males. Average compliance was 87%. One hundred patients (53.76%) had injection compliance of ≥90%. Amongst these patients, 74%, 68%, and 77% top-scored (5/5) the technical features of hidden needle, skin sensor, and pre-set dosing, respectively, compared to top scores by 39%, 34%, and 51% patients in the <90% compliance group (*p*-value <0.05). Similarly, a statistically significant difference was observed between the groups (*p*-value <0.05) in the perception of the usefulness of the tracking features such as display of history of injected doses (78% vs. 47.7%), a reminder for medicine remaining (46% vs. 23.3%) and battery power indicator (48% vs. 20.9%). Personal screen messages were associated with higher compliance while the requirement to keep the device in the fridge was reported as the most inconvenient feature by 56% of patients in the higher compliance group as against 39.5% in the lower compliance group (*p*-value <0.05). There was no statistically significant difference in the intensity of pain reported in the two compliance groups.

**Conclusion:**

Our study showed that there is a statistically significant association between better perception of device features and higher compliance.

## Introduction

The availability of recombinant human growth hormone (rhGH) has made GH treatment for short stature widely available (1). Growth hormone treatment has a wide list of indications, some of which have received approval from the Food and Drug Administration or European Medicines Agency. These include GH deficiency (GHD), Turner syndrome, short stature related to a birth size small for gestational age (SGA), idiopathic short stature, and growth failure in pre-pubertal children due to chronic renal insufficiency (CRF) (2–4).

GH therapy has been demonstrated to improve short-term growth and adult height in approved indications (5). However, considerable variability in response has been noted depending on the age at the start of therapy, GH dose, genetic conditions, concomitant illness, and compliance (3, 6). As with any chronic long-term treatment, rhGH treatment is burdened with suboptimal adherence, especially in a pediatric population (7). Factors affecting adherence to GH therapy include the patients' preference for the GH delivery device, its simplicity, and convenience, as well as appropriate education and technical training (8). Daily subcutaneous GH injections can create a significant treatment load, negatively influencing adherence to therapy (2). Adherence to the recommended treatment regimen is important for successful outcomes with rhGH therapy to ensure that patients reach their target height (9). Low adherence is also associated with less favorable clinical outcomes and increased healthcare costs (10).

Several strategies have been proposed to improve adherence. These include improving device simplicity, convenience, and education and training of patients and parents (2). A recent survey of patients, parents, physicians and nurses with experience in the administration of rhGH suggested that reliability, ease of use, lack of pain during injection, safety in use and storage, and a minimum number of steps before injection preparation, were all important factors (10). In addition, a good tracking system to objectively monitor treatment adherence was considered extremely important by the treating physicians (10). Precise information on treatment adherence allows the clinician to exclude poor adherence as a possible reason for sub-optimal growth response, driving further treatment adjustment (2). Multiple long-acting GH (LAGH) preparations are also currently being developed in an attempt to decrease GH injection frequency from daily to weekly, biweekly, or monthly, thereby attempting to improve adherence (11).

EasyPod™ is an electronic auto-injector device that is equipped for adherence monitoring. It has several features including pre-set dosing, adjustable injection settings, and monitoring of adherence using an injection log that records injection history, which can be accessed by patients and clinicians to monitor adherence. The device is equipped with other functions such as screen reminders for battery life, medication cartridge filling, and the number of medication doses left to encourage better compliance. In addition, the device has specific features to encourage children to use it, including protective skin covers, colorful device screen outliners, customized screen messages, and screen photographs of the patients' choice. These features are intended to create a bond between children and their devices and improve compliance.

The present study was carried out to assess patient satisfaction with the technical and tracking features of the EasyPod™ delivery device and its association with compliance. We also aimed to explore the most preferred personal features of the device, patients' scoring of pain severity, and drawbacks of the device. This users' feedback will enable the application of various improvement strategies on the device, encouraging better utilization with the ultimate aim of improving treatment adherence.

## Materials and Methods

### Structure of the Study

This was a questionnaire-based, multicenter, survey study conducted from the 1st of March to the 30th of June 2020. The initial study was planned to be carried out at eight centers from three countries (United Arab Emirates, Oman, and Saudi Arabia), but due to the lack of compliance data from two centers, the final data analysis was carried out with data from six centers. A total of 186 children and adolescents diagnosed with growth disorders in the age group of 3–18 years and receiving rGH (Saizen®, Merck Serono International SA, Geneva, Switzerland) through the EasyPod™ (Merck Serono International SA, Geneva, Switzerland) device were enrolled in the survey. All enrolled subjects were on GH treatment for a variable period, with a mean (±SD) duration of 3.74 (±2.9) years and were using the EasyPod™ device only. Participants above 12 years mostly self-injected, while younger children were helped by their parents/carers to inject. Informed consent was obtained from the participants and/or their parents. The patients' compliance to injectable therapy was reviewed by downloading the data recorded in the device and is taken as a percentage over the latest 3–6 months period of use.

The aim of the study and details of the questionnaire were explained to the participants by the study team consisting of pediatric endocrinologists and endocrine nurses. The study was approved by local institutional review boards.

### Scoring Systems Within the Questionnaire

The questionnaire was designed by co-authors who formed a focus group to assess users' satisfaction and perception of use. It was validated for use by multiple trials in the clinic with staff and users before commencing the study. It included questions on five main areas related to the device (Appendix).

1. Patient satisfaction with the technical features (Q1)
2. Patients' views on tracking features and compliance support (Q2-3)
3. Patients' preferred personalized feature (Q4)
4. Patient scoring of pain on device use (Q5)
5. Experience on device drawbacks (Q6)

The Question on satisfaction with technical features (Q1) of the device was scored on a 5-point scale, with 1 being un-useful, 2 less useful, 3 neutral, 4 useful and 5 being very useful. Tracking features were assessed in 2 questions. Question 2 was scored on a 5 points scale (as above) and Question 3 was designed to have an answer of either “Yes,” “No,” or “Neutral.” Inquiry on preferred personalized device features (Q4) was assessed through a multiple-choice question in which a list of personalized features was given to choose from. Pain intensity was scored on a 5- point scale, with 1 being completely painless, 2 minimal, 3 mild pain, 4 moderate pain and 5 being very painful (Q5). Views on device drawbacks were enquired about in a multiple-choice question which users scored for the most appropriate answer (Q6).

### Endpoints of the Survey

The primary endpoint of the survey was to assess patient satisfaction with the technical and tracking features of the EasyPod™ delivery device and its association with compliance. The secondary endpoint was to explore patients' preferred personalized device features, pain experience, and device drawbacks. Device compliance of 85% is considered to be satisfactory as per the literature (9, 12). However, considering the overall high compliance in our cohort, we considered compliance of 90% or more as the cut-off level to perform a segmental analysis on this proportion of patients.

### Statistics and Data Analysis

Standard descriptive statistics and demographic and primary data analysis was carried out in 186 patients. The proportion analysis of patients who rated the automated dose delivery and tracking features as being “very useful” and helpful in tracking and increasing compliance was summarized using frequency count. Mean (± SD) values were calculated for the baseline characteristics of patients' ages and duration of treatment with GH. The data was divided into two subgroups based on compliance (<90% compliance and ≥90% compliance). For ordinal data obtained from Q1, Q2, and Q5, the Kruskal-Wallis H test (sometimes also called the “one-way ANOVA on ranks”) was used to determine if there are statistically significant differences between the two groups (compliance <90% vs. ≥90%) to an independent variable on a continuous dependent variable. For categorical data (Q3, Q4, Q6), univariate logistic regression analysis was used for statistically significant differences between two or more groups (compliance <90% vs. ≥90%).

## Results

### Subjects and Diagnoses

A total of 186 subjects were enrolled from six centers in three countries (Center 1: 32, Center 2: 12, Center 3: 17, Center 4: 65, Center 5: 40, Center 6: 20). The mean age ± SD of the enrolled patients was 11.8 ± 2.76 years. There were 117 males and 65 females in the study. Gender information was missing for four patients. The mean ± SD duration of treatment with growth hormone was 3.74 ± 2.9 years. The mean percentage of compliance recorded was 87% (range: 50–100%). A hundred patients (53.76%) had injection compliance of ≥90%; mean compliance in this group was 95% (range: 90–100%). The average duration of use of the device in these patients was 3.49 ± 2.98 years compared to 3.56 ± 2.33 in the 86 patients with <90% compliance (mean compliance of 79%; range: 50–89%). Twenty-nine patients had 100% compliance and the average duration of use in these patients was 2.57 ± 2.76 years. The majority of patients were diagnosed with GH deficiency (48.9%) of which 6 patients (6.6%) had panhypopituitarism. Other diagnoses were idiopathic short stature (21.5%), small for gestational age (15.6%), chronic renal failure (3.2%), and Turner's syndrome (3.2%). Other conditions included were Noonan syndrome (2.7%), skeletal dysplasia (2.2%), Fanconi Bickel syndrome (1.1%), rheumatoid disease (0.5%), and osteogenesis imperfecta (0.5%). Data on diagnosis was missing for one patient (Table 1).

**Table 1:** Baseline demographic profile (*N* = 186).

Mean (±SD) age	11.8 (±2.76)
Sex ratio (M:F)^a^	117:65
GH indication^b^ *N* (%)	
GH deficiency *N* (%)	91 (48.9)
Panhypopituitarism	6 (6.6%)^c^
Idiopathic short stature *N* (%)	40 (21.5)
Small for gestational age *N* (%)	29 (15.6)
Chronic renal failure *N* (%)	6 (3.2)
Turners syndrome *N* (%)	6 (3.2)
Noonan syndrome *N* (%)	5 (2.7)
Skeletal dysplasia *N* (%)	4 (2.2)
Fanconi Bickel syndrome *N* (%)	2 (1.1)
Rheumatoid disease *N* (%)	1 (0.5)
Osteogenesis imperfect *N* (%)	1 (0.5)
Total	185
Duration of GH indication in years [Mean (±SD)]	3.74 (±2.9)
Average compliance (%)	87%
Mean compliance (<90%) (Range)	79% (50–89%)
Mean compliance (≥90%) (Range)	95% (90–100%)

a *Gender information is missing for four patients*.

b *Indication data is missing for one patient*.

c *Proportion of patients with GH deficiency*.

### Scoring of Automated Dose Delivery Features of the Device

The data set for the primary endpoint analysis comprised of 186 patients. The technical features of the device were scored as follows: 74% of patients in the higher compliance group of ≥90% reported the hidden needle feature to be “very useful” compared to 45.3% in the lower compliance group. Similarly, the skin sensor and pre-set dosing facility were scored as “very useful” by 68 and 77% of patients, respectively, in the higher compliance group as compared to 39.5 and 59.3% in the lower compliance group (Table 2). A Kruskal-Wallis H test showed that there was a statistically significant difference in perception regarding the usefulness of the hidden needle feature between the two compliance groups (<90% vs. ≥ 90%, [χ^2^ (1) = 18.943, *p* **<** 0.001]), with a mean rank of 77.31 in the <90% compliant group and 107.42 in the ≥90% compliant group. Similarly, patients in the ≥90% compliance group were statistically significantly more satisfied with the skin sensor feature [mean rank of 78.80 in the <90% compliance group and 106.14 in the ≥90% compliance group; χ^2^ (1) = 14.756, *p* **<** 0.001], and the pre-set dosing feature [mean rank of 84.56 in the <90% compliance group and 101.18 in the ≥ 90% compliance group; χ^2^ (1) = 6.616, *p* **=** 0.010] compared to patients in the <90% compliance group.

**Table 2:** Comparison between compliance groups for the usefulness of automated dose delivery and tracking features (*N* = 186).

		Compliance (<90%)^*^ **(*n =* 86)**	Compliance (≥90%)^*^ **(*n =* 100)**	Total^*^ **(*n =* 186)**	**χ^2^ test** ** statistics**	***P*-value**
**Automated dose delivery features**						
Hidden needle that auto-injects the medicine	Un-useful	0 (0.0)	0 (0.0)	0 (0.0)	18.943	<0.001
	Less useful	9 (10.5)	1 (1.0)	10 (5.4)		
	Neutral	16 (18.6)	7 (7.0)	23 (12.4)		
	Useful	22 (25.6)	18 (18.0)	40 (21.5)		
	Very Useful	39 (45.3)	74 (74.0)	113 (60.8)		
Skin sensor that helps with injection technique	Un-useful	0 (0.0)	0 (0.0)	0 (0.0)	14.756	<0.001
	Less useful	4 (4.7)	0(0.0)	4 (2.2)		
	Neutral	15 (17.4)	11 (11.0)	26 (14.0)		
	Useful	33 (38.4)	21 (21.0)	54 (29.0)		
	Very Useful	34 (39.5)	68 (68.0)	102 (54.8)		
Preset dosing so no daily dialing is required	Un-useful	0 (0.0)	1 (1.0)	1 (0.50)	6.616	0.010
	Less useful	0 (0.0)	0 (0.0)	0 (0.0)		
	Neutral	15 (17.4)	8 (8.0)	23 (12.4)		
	Useful	20 (23.3)	14 (14.0)	34 (18.3)		
	Very Useful	51 (59.3)	77 (77.0)	128 (68.8)		
**Tracking features**						
History of injected and missed doses	Un-useful	14 (16.3)	2 (2.0)	16 (8.6)	23.266	<0.001
	Less useful	4 (4.7)	0 (0)	4 (2.2)		
	Neutral	14 (16.3)	6 (6.0)	20 (10.8)		
	Useful	13 (15.1)	14 (14.0)	27 (14.5)		
	Very Useful	41 (47.7)	78 (78.0)	119 (64.0)		
Amount of medicine left in cartridge reminding patients of time to change cartridge	Un-useful	7 (8.1)	2 (2.0)	9 (4.8)	17.908	<0.001
	Less useful	15 (17.4)	5 (5.0)	20 (10.8)		
	Neutral	24 (27.9)	19 (19.0)	43 (23.1)		
	Useful	20 (23.3)	28 (28.0)	48 (25.8)		
	Very Useful	20 (23.3)	46 (46.0)	66 (35.5)		
Battery power left	Un-useful	15 (17.4)	4 (4.0)	19 (10.2)	20.375	<0.001
	Less useful	9 (10.5)	9 (9.0)	18 (9.7)		
	Neutral	26 (30.2)	14 (14.0)	40 (21.5)		
	Useful	18 (20.9)	25 (25.0)	43 (23.1)		
	Very Useful	18 (20.9)	48 (48.0)	66 (35.5)		

* *n (%)*.

### Tracking Features

Among the tracking features, a statistically significant higher proportion of patients (78%) in the ≥90% compliance group reported the feature of the history of injected and missed doses to be “very useful” as compared to 47.7% in the <90% compliance group (<90% vs. ≥90%, [χ^2^ (1) = 23.266, *p* **<** 0.001]) (Table 2), with a mean rank of 75.92 in the <90% compliance group and 108.62 in the ≥90% compliance group.

The feature of medicine left in cartridge reminder and the battery power indicator was rated to be “very useful” by 46 and 48%, respectively, by patients in the higher compliance group (Table 2). This was significantly higher than the patients in the lower compliance group (23.3 and 20.9%) (*p* < 0.001). A Kruskal-Wallis H test showed that there was a statistically significant difference in perception regarding the cartridge change notification feature between the different compliance groups [ <90% vs. ≥90%; χ^2^ (1) = 17.908, *p* **<** 0.001], with a mean rank of 76.18 in the <90% compliance group and 108.40 in the ≥90% compliance group. Similarly, a Kruskal-Wallis H test showed that there was a statistically significant difference in perception regarding the battery power left notification feature between the different compliance groups [ <90% vs. ≥90%; χ^2^ (1) = 20.375, *p* **<** 0.001], with a mean rank of 74.96 in the <90% compliance group and 109.44 in the ≥90% compliance group.

Among highly compliant patients, 80% found the tracking features of the device to be useful in tracking missed doses and in encouraging their child to be more compliant. However, a Chi-square test showed that this number was not statistically significantly higher [χ^2^ (1) = 1.183, *p* **=** 0.277] than that in the lower compliance group (<90%).

### Personalized Features

A Chi-square test was not statistically significant between compliance groups in choice of personalized device features such as colorful covers [χ^2^ (1) = 1.151, *p* **=** 0.562], device skins [χ^2^ (1) = 0.058, *p* **=** 0.810] and welcome picture [χ^2^ (1) = 0.786, *p* **=** 0.375]. However, the personal screen message feature was significantly [χ^2^ (1) = 4.212, *p* **=** 0.040] associated with a higher (≥90%) compliance status (Table 3).

**Table 3:** Comparison between compliance groups for the preferred personalized device features (*N* = 186).

	Compliance (<90%)^*^ **(*n =* 86)**	Compliance (≥90%)^*^ **(*n =* 100)**	Total^*^ **(*n =* 186)**	**χ^2^ test** ** statistics**	***P*-value**
Colorful covers	22 (25.6)	22 (22.0)	44 (23.7)	1.151	0.562
Device skins	16 (18.6)	20 (20.0)	36 (19.4)	0.058	0.810
Welcome picture	20 (23.3)	18 (18.0)	38 (20.4)	0.786	0.375
Personal screen message	44 (51.2)	66 (66.0)	110 (59.1)	4.212	0.040

* *n (%)*.

### Pain on Injection

In the higher compliance group, 48% of patients reported no pain experience on using the device (score of 1/5) and none reported a score of 5/5 (indicative of severe pain). Further, 49% of patients reported minimal to mild pain (scores of 2/5 and 3/5) in this group. However, a Kruskal-Wallis H test showed that there was no statistically significant difference in pain scores between the two compliance groups [ <90% vs. ≥90%; χ^2^ (1) = 1.359, *p* **=** 0.244], with a mean rank of 97.10 for compliance <90% and 88.55 for compliance ≥90% (Table 4).

**Table 4:** Comparison between compliance groups for the perception of pain with EasyPod^TM^ device (*N* = 186).

		Compliance (<90%)^*^ **(*n =* 86)**	Compliance (≥90%)^*^ **(*n =* 100)**	**Total** ** (*n =* 186)**	**χ^2^ test** ** statistics**	***P*-value**
How painful do you find injection with EasyPod	Complete Painless	36 (41.9)	48 (48.0)	84 (45.2)	1.359	0.244
	Minimal Pain	23 (26.7)	30 (30.0)	53 (28.5)		
	Mild Pain	25 (29.1)	19 (19.0)	44 (23.7)		
	Moderate Pain	1 (1.2)	2 (2.0)	3 (1.6)		
	Very Painful	0 (0.0)	0 (0.0)	0 (0.0)		

* *n (%)*.

### Most Inconvenient Feature

The requirement of keeping the device in the fridge was reported as the most inconvenient feature in the higher compliance group by 56% of patients as compared to 39.5% patients in the lower compliance group (*p*-value <0.05). However, there was no statistically significant association between choice of others features such as special batteries [χ^2^ (1) = 3.395, *p* **=** 0.183], special needles [χ^2^ (1) = 3.145, *p* **=** 0.208] and heavy device [χ^2^ (1) = 4.555, *p* **=** 0.103] as being the most inconvenient, and compliance status (Table 5).

**Table 5:** Comparison between compliance groups for the most inconvenient feature of the device.

	Compliance (<90%)^*^ **(*n =* 86)**	Compliance (≥90%)^*^ **(*n =* 100)**	Total^*^ **(*n =* 186)**	**χ^2^ test** ** statistics**	***P*-value**
It has to be kept in the fridge	34 (39.5)	56 (56.0)	90 (48.4)	13.971	0.003
Special batteries	20 (23.3)	18 (18.0)	38 (20.4)	3.395	0.183
Special needles	22 (25.6)	27 (27.0)	49 (26.3)	3.145	0.208
Heavy device	39 (45.3)	34 (34.0)	73 (39.2)	4.555	0.103

* *n (%)*.

An analysis to evaluate the association between the scores and age of the patient did not show any statistically significant difference ( ≤ 10 years and >10 years).

## Discussion

In the present study, patients and their parents or caregivers were surveyed over 4 months to evaluate patients' perception of various functionalities of the device and its impact on compliance. The study demonstrated a high GH treatment compliance in children and adolescents (87%), which is more than the minimum percentage recommended to be considered as optimal adherence to hGH administration (85%) (9, 12). The present study results demonstrated that higher scores of patient satisfaction with the technical and the tracking features of the EasyPod™ delivery device were significantly associated with higher compliance.

Over the years, several attempts have been made to understand patient needs and improve device design. The traditional syringes with needles have been replaced with more innovative user-friendly devices that include injection pens, self-injection pens, needle-free devices, and electronic devices with the potential to improve adherence. Adherence to treatment plays a vital role in the overall clinical outcomes of GH therapy (3). In a large multinational, observational study enrolling children receiving GH treatment through the EasyPod™ device, conducted between 2010 and 2016, it was observed that better adherence to treatment resulted in significant positive growth outcomes (13). Poor compliance resulted in significantly lower growth rates in comparison to patients who missed fewer doses (14).

Studies have suggested that decreased adherence may occur with increasing duration of treatment due to lack of enthusiasm or motivation about adhering to treatment compared with those new users, who may be more diligent (15). In the study by Koledova et al., median adherence rates were high (94%) in the first year of treatment which gradually decreased over follow-up, but a majority of patients maintained ≥80% of adherence over 3 years of treatment (13). In our study too, the average duration of use in patients who had 100% compliance was 2.57 ± 2.76 years, while the entire study population which reported an average duration of use of 3.74 ± 2.9 years showed a lower compliance rate of 87%.

Most common device-related features that impact adherence levels as reported by parents include the product delivery system, its simplicity, convenience, and ease of use, and availability of appropriate training in the administration technique (8, 16). In a study to compare the optimum device for GH administration, a vial combined with an auto-injector or a pen injection system using a cartridge was compared. The study showed that patients preferred auto-injection devices over manual insertion of a needle (17). Dahlgren et al. showed that the auto-injector and skin sensor features of the EasyPod™ device help in increasing the accuracy of auto-injection (18). In an observational 3 month survey with children receiving r-hGH through EasyPod™, 82.5% of participants found the electronic auto-injector easy/very easy to prepare, 92.4% of patients said that the device was easy/very easy to use, 85.0% rated the duration of injection as short/very short and 61.5% reported experiencing no pain when injecting with the electronic auto-injector (19). In our study too, patients were significantly more satisfied with the automated dose delivery features of the hidden needle, skin sensor, and pre-set dosing of the EasyPod™ device and reported a higher compliance rate of ≥90%.

The EasyPod™ device also has improvised display features. The display screen is larger compared to other devices and features high contrast and resolution to be easily read. Studies have shown that the provision of clear instructions affects patient preference for an injection device (20). Our study also demonstrated a high preference for personal screen messages by participants which was associated with higher compliance as well.

In a survey of another autoinjector GH administration device (Sure Pal™), patients or their caregivers rated the dose-memory function as being very helpful/helpful (66.2%). The EasyPod™ device also has an inbuilt electronic adherence monitoring system that provides physicians with personal adherence data. A significantly higher number of patients in the high compliance group of ≥90% found the downloadable and tracking feature of the device to be useful in tracking injected and missed doses. Various other functions such as screen reminders for battery life and medication cartridge filling are also associated with higher compliance.

Stanhope et al., showed that patients experienced less pain with the auto-injector as compared to the pen and also reported less wastage of growth hormone (17). The high patient acceptance and satisfaction of the device in our study are aligned with a favorable safety profile and a high proportion of subjects reporting no pain (45.2%). None of the patients surveyed reported severe pain, and only 3 reported moderate pain as per the pain scale used. We hypothesize that the hidden needle feature of the device could be a factor in reducing the pain as the feature was rated highly by the majority of the subjects. It is reported that the hidden needle feature is less likely to make the patient anxious and consequently less sensitive toward pain (21). However, there is no statistically significant association in the perception of pain with EasyPod™ and higher compliance.

It can be anticipated that parents well-informed on the diagnosis, and the modalities and problems related to treatment might be more motivated to do the best for their children, to know all possibilities offered by the device. This would once again underline the importance of accurate information given to the parent at the beginning of treatment in order to obtain a better result.

The current study results are in agreement with previous studies using smaller sample sizes and shorter duration and indicate a high level of patient acceptance of the electronic auto-injector for the daily administration of GH. The features of EasyPod™ are considered useful in routine practice and a majority of participants express a desire to continue using the device (22). Patient feedback on drawbacks and pain scores can also provide a basis for improving the technical features and better utilizing the comfort setting for further improving compliance.

A limitation of the study was the design being a cross-sectional open-label survey. The comparison between treatment-naïve and treatment-experienced could not be done. A controlled longitudinal design can be envisaged in the future.

## Conclusions

Patients in the ≥90% compliance group were more satisfied with the automated dose delivery and tracking features of the EasyPod™ device in comparison to those with lesser compliance. Formal education of the device's advanced technical features may further improve satisfaction and ensure injection compliance.

## Data Availability Statement

The original contributions presented in the study are included in the article/Supplementary Material, further inquiries can be directed to the corresponding author/s.

## Ethics Statement

The studies involving human participants were reviewed and approved by Sheikh Shakhbout Medical City Research Ethics Committee. Written informed consent to participate in this study was provided by the participants' legal guardian/next of kin.

## Author Contributions

AD designed the study, collected site data, and liaised between co-authors. All authors contributed equally to the development of the manuscript.

## Conflict of Interest

This publication received funding from Merck Serono Middle East FZ-LTD. The funder was not involved in the study design, collection, analysis, interpretation of data, the writing of this article, or the decision to submit it for publication. This publication is done via an unrestricted medical writing grant by Merck Serono Middle East FZ-LTD, an affiliate of Merck KGaA, Darmstadt, Germany. The authors declare that the research was conducted in the absence of any commercial or financial relationships that could be construed as a potential conflict of interest.

## Publisher's Note

All claims expressed in this article are solely those of the authors and do not necessarily represent those of their affiliated organizations, or those of the publisher, the editors and the reviewers. Any product that may be evaluated in this article, or claim that may be made by its manufacturer, is not guaranteed or endorsed by the publisher.
