# Supplementary material for: Patients' Perception of the Use of the EasyPod™ Growth Hormone Injector Device and Impact on Injection Adherence: A Multi-Center Regional Study
Source: Front Pediatr. 2022 Feb 28;10:839278. doi: 10.3389/fped.2022.839278 (PMC8918687; doi:10.3389/fped.2022.839278)
Supplement: Supplementary file 1 [file Data_Sheet_1.PDF]

Questionnaire for assessing patient satisfaction with the EasyPod™ device

1) **How useful do you find the following automated dose delivery features (scale of 1-5 with 1 being very un-useful to 5 very useful):**
   1- Hidden needle that auto-injects the medicine
   2- Skin sensor that helps with injection technique
   3- Preset dosing so no daily dialing is required
2) **How useful do you find the following tracking features (scale of 1-5 with 1 being very un-useful to 5 very useful):**
   1- History of injected and missed doses
   2- Amount of medicine left in cartridge reminding patients of time to change cartridge
   3- Battery power left
3) **What is your views on the downloadable and tracking function of the Easypod (Yes, No, Neutral)**
   1- Good feature that helps us tracking missed doses
   2- Useful to encourage my child to be more compliant
4) **What personalized device feature do you prefer?**
   1- Colorful covers
   2- Device skins
   3- Welcome picture
   4- Personal screen message
5) **How painful do you find injection with EasyPod? (scale of 1-5 with 1 being painless to 5 very painful)**
6) **What is the most inconvenient feature you face with the device?**
   1- It has to be kept in the fridge
   2- Special batteries
   3- Special needles
   4- Heavy device
